# Processing speed is related to striatal dopamine transporter availability in Parkinson's disease

**DOI:** 10.1016/j.nicl.2020.102257

**Published:** 2020-04-21

**Authors:** Chris Vriend, Tim D. van Balkom, Corné van Druningen, Martin Klein, Ysbrand D. van der Werf, Henk W. Berendse, Odile A. van den Heuvel

**Affiliations:** a Amsterdam UMC, Vrije Universiteit Amsterdam, Psychiatry, Amsterdam Neuroscience, De Boelelaan 1117, Amsterdam, the Netherlands; b Amsterdam UMC, Vrije Universiteit Amsterdam, Anatomy and Neurosciences, Amsterdam Neuroscience, De Boelelaan 1117, Amsterdam 1007 MB, the Netherlands; c Amsterdam UMC, Vrije Universiteit Amsterdam, Medical Psychology, Amsterdam Neuroscience, De Boelelaan 1117, Amsterdam, the Netherlands; d Amsterdam UMC, Vrije Universiteit Amsterdam, Neurology, Amsterdam Neuroscience, De Boelelaan 1117, Amsterdam, the Netherlands

**Keywords:** Parkinson, Cognition, Nuclear imaging, Dopamine, Serotonin, Striatum

## Abstract

**Background:**

Parkinson's disease (PD) affects the integrity of the dopamine and serotonin system, and is characterized by a plethora of different symptoms, including cognitive impairments of which the pathophysiology is not yet fully elucidated.

**Objectives:**

Investigate the role of the integrity of the dopaminergic and serotonergic system in cognitive functioning in early-stage PD using Single Photon Emission Computed Tomography (SPECT) combined with the radiotracer ^123^I-N-ω-fluoropropyl-2β-carbomethoxy-3β-(4-iodophenyl)nortropane (^123^I-FP-CIT).

**Methods:**

We studied the association between cognitive functions and dopamine transporter (DAT) availability in the caudate nucleus and putamen – as a proxy for striatal dopaminergic integrity – and serotonin transporter (SERT) availability as a proxy for serotonergic integrity in the thalamus and hippocampus using bootstrapped multiple regression. One-hundred-and-twenty-nine (129) PD patients underwent a ^123^I-FP-CIT SPECT scan and a neuropsychological assessment.

**Results:**

We showed a positive association between DAT availability in the head of the caudate nucleus and the Stroop Color Word Task – card I (reading words; *β* = 0.32, *P* = 0.001) and a positive association between DAT availability in the anterior putamen and the Trail Making Test part A (connecting consecutively numbered circles; *β* = 0.25, *P* = 0.02). These associations remained after adjusting for motor symptom severity or volume of the region-of-interest and were most pronounced in medication-naïve PD patients. There were no associations between cognitive performance and SERT availability in the thalamus or hippocampus.

**Conclusions:**

We interpret these results as a role for striatal dopamine – and its PD-related decline – in aspects of processing speed.

## Highlights

• Cognitive problems are common in Parkinson's disease.
• We investigated their association with dopaminergic and serotonergic degeneration.
• Lower striatal dopamine was associated with reduced processing speed.
• There was no association with serotonin availability.
• Striatal dopamine and Parkinson-related degeneration plays a role in processing speed.

## 1 Introduction

Cognitive impairments are common in Parkinson's disease (PD), with cognitive impairment already present in up to twenty-five percent of patients around the time of diagnosis (Muslimovic et al., 2005; Weintraub et al., 2015). Tasks that require cognitive flexibility, (divided) attention or that are time-sensitive are especially affected in PD (Muslimovic et al., 2005; Pagonabarraga and Kulisevsky, 2012). Furthermore, up to eighty percent of patients will eventually develop PD dementia (PDD) (Litvan et al., 2011). The incidence of cognitive impairments increases steadily with progression of the disease which implies a role of the spreading PD pathology in its etiology. Indeed, the severity of post-mortem cortical Lewy body pathology has been shown to be associated with increased cognitive impairments (Halliday et al., 2014).

The integrity of dopaminergic projections can be approximated in vivo by measuring the availability of striatal presynaptic dopamine transporters (DAT) using radiotracers, such as ^123^I-N-ω-fluoropropyl-2β-carbomethoxy-3β-(4-iodophenyl)nortropane (^123^I-FP-CIT) in combination with single photon emission computed tomography (SPECT). Using such radiotracers, studies have shown lower striatal DAT binding, indicative of more dopamine denervation (Scherfler et al., 2007), in PD patients with cognitive impairments compared with those without (Chung et al., 2018; Ekman et al., 2012; Schrag et al., 2017). DAT availability is also associated with cognitive performance (Chung et al., 2018; Siepel et al., 2014; Nobili et al., 2010; Jokinen et al., 2009) and task-related brain activation (Trujillo et al., 2015; Vriend et al., 2015). Dopamine is the primary modulator of the cortico-striato-thalamo-cortical (CSTC) circuits (Groenewegen and Uylings, 2010). Especially within the associative CSTC circuit, involving connections between the striatal regions and prefrontal cortex, dopamine deficiencies are associated with cognitive impairments (Chung et al., 2018; Ekman et al., 2012; Siepel et al., 2014; Nobili et al., 2010; Jokinen et al., 2009).

Other neurotransmitters are also involved in cognition, such as acetylcholine and serotonin (Roy et al., 2016; Ballinger et al., 2016; Cowen and Sherwood, 2013; Svob Strac et al., 2016). These neurotransmitters also play a role in the pathophysiology of PD but has so far received less scientific attention (Politis and Niccolini, 2015; Gratwicke et al., 2015; Pagano et al., 2017). The radiotracer ^123^I-FP-CIT has, in addition to its high affinity for the DAT, also modest affinity for the serotonin transporter (SERT). Due to the differential expression patterns of DAT and SERT, ^123^I-FP-CIT binding in the striatal regions predominantly reflects DAT availability, while ^123^I-FP-CIT binding in extrastriatal regions, such as the hippocampus and (hypo)thalamus, represents SERT availability (Booij et al., 2007). A single ^123^I-FP-CIT SPECT scan can therefore be used to simultaneously measure DAT and SERT availability, as demonstrated previously (Joling et al., 2018, 2017; Roselli et al., 2010).

The SERT-rich hippocampus and thalamus are important brain regions for memory and for executive functions, attention and (working) memory, respectively (Varnas et al., 2004; Fama and Sullivan, 2015; Saalmann and Kastner, 2015; Eichenbaum, 2017). Patients with dementia with Lewy bodies (DLB) showed reduced ^123^I-FP-CIT SPECT binding in the thalamus compared with both PD patients and healthy controls (Pilotto et al., 2019). Although DLB is characterized by severe cognitive deficits and thought to share an underlying pathophysiology with PD, the clinical significance of this finding is currently unclear. In non-PD samples, no relationship was observed between SERT availability and cognitive performance in young adult healthy participants with normal cognition (Burke et al., 2011), while another found reduced SERT availability in patients with mild cognitive impairment (MCI) in various brain regions, including the thalamus, that correlated with worse memory performance (Smith et al., 2017). This correlation was not observed in cognitively normal controls, suggesting that associations with SERT are only observed under pathological conditions (Smith et al., 2017).

In this study we investigated the association between striatal DAT and extrastriatal SERT availability and cognitive function in PD patients to provide further evidence that monoamine deficiencies are related to PD-related cognitive impairments. Based on previous research we hypothesized a positive association between cognitive performance, in particular executive functions, attention and working memory, and striatal DAT availability, specifically in the caudate nucleus (Chung et al., 2018; Ekman et al., 2012; Siepel et al., 2014; Nobili et al., 2010; Jokinen et al., 2009). Based on the study by Smith and colleagues (Smith et al., 2017) in patients with MCI we hypothesized a positive correlation between thalamic and hippocampal SERT availability and cognitive performance in PD, specifically memory and attention.

## 2 Patients and methods

### 2.1 Participants

**Fig. 1:**
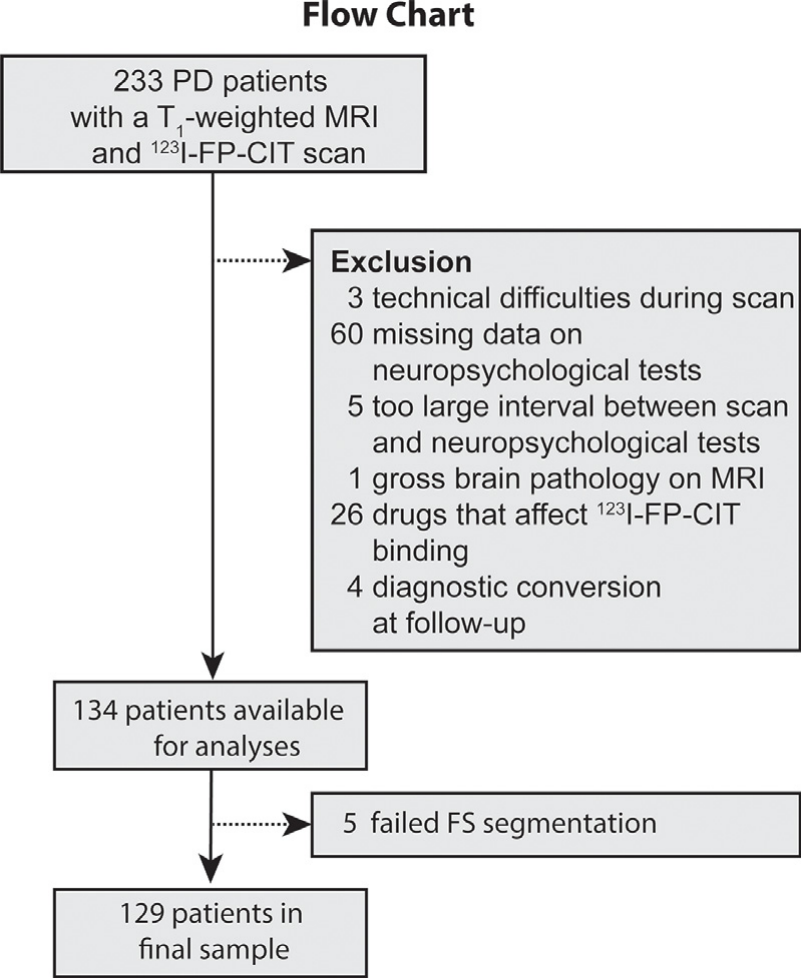
Flowchart of the excluded patients in this study.

PD patients were selected from a database of consecutive cases that visited the outpatient clinic for movement disorders of the neurology department of the Amsterdam UMC, location VU University Medical Center (Amsterdam, The Netherlands) between May 2008 and February 2017. Overlapping samples derived from this cross-sectional database have also been used for previous investigations into non-motor symptoms of PD (e.g. Vriend et al., 2015; Joling et al., 2018; Vriend et al., 2016; Vriend et al., 2014a). Diagnosis of PD was established by movement disorder specialists according to the UK PD Society Brain Bank criteria (Hughes et al., 1992). Diagnosis was confirmed in 89% of the included patients after at least two year follow-up. The others were lost to follow-up. Patients were eligible for this study when they were administered a ^123^I-FP-CIT SPECT scan, a T_1_-weighted magnetic resonance imaging (MRI) scan and a neuropsychological assessment. Patients in our center are administered these scans and the assessment as part of the diagnostic work-up. We excluded patients on selective serotonin reuptake inhibitors (SSRIs), because SSRIs can displace ^123^I-FP-CIT from the DAT and SERT (Booij et al., 2007). See Fig. 1 for a flowchart. In accordance with the Declaration of Helsinki, all included patients gave written informed consent to use their clinical and neuroimaging data for scientific purposes, and the study was approved by the local medical ethics committee.

### 2.2 Clinical and neuropsychological measures

We evaluated motor symptom severity with the Unified PD Rating Scale, Section 3 (UPDRS-III) (Fahn and Elton, 1987). Patient that were on dopamine replacement therapy were measured in their ‘ON’ phase during all assessments and the SPECT scan. For these patients we calculated the levodopa equivalent daily dose (LEDD) to standardize the medication dosages (Olde Dubbelink et al., 2013). Severity of depressive symptoms was assessed using the Beck Depression Inventory (BDI). We scored education level according to the Dutch Verhage scale (Verhage, 1964) that ranges from 1 – *primary school not finished*, to 7 – *university or higher*. We administered the Mini-mental state examination (MMSE) (Folstein et al., 1975), as a measure of global cognition. The neuropsychological assessment comprised the Stroop Color Word Test (SCWT), the Trail Making Test (TMT), the digit span subtest of the Wechsler Adult Intelligence Scale (WAIS)-III, the Dutch version of the Rey Auditory Verbal Learning Test (RAVLT) and the delayed recall of the Rey Complex Figure Test (RCFT). These neuropsychological tests were performed to assess a range of cognitive functions: (*1) executive functions:* we used the SCWT color-word card (SCWT-III) time corrected for color-only card (SCWT-II) time as a measure of interference control. The TMT task B time corrected for task A time was used as a measure of set-shifting; (*2) attention and processing speed:* we used the time on the word reading card of the SCWT (SCWT-I) and the time on the TMT card A (TMT-A; connecting consecutively numbered circles) as measures of attention and processing speed. We used the total score on the forward digit span of the WAIS-III as a measure of attention; *(3) working memory:* the WAIS-III backward digit span score was used as a measure of working memory; *(4) episodic memory:* verbal episodic memory was measured with the delayed recall score of the RAVLT. We measured visuospatial episodic memory with the delayed recall of the RCFT. We did not include the SCWT-II as a separate measure of attention and processing speed in our analyses as this was highly correlated with the SCWT-I. Likewise, we did not use the direct recall score of the RAVLT as separate measure in our analyses given the high correlation with the delayed recall score. The RAVLT recognition trial was excluded from the analyses due to ceiling effects. All neuropsychological assessments were performed within two weeks of the ^123^I-FP-CIT scan. All scores on the neuropsychological tests were converted to standardized T-scores (with a mean of 50 and standard deviation of 10) or percentiles to adjust for age, sex and/or educational level, using the appropriate Dutch norm scores (Schmand et al., 2012). See Table 1.

**Table 1:** Cognitive tests per domain and associations with regions of interest.

### 2.3 ^123^I-FP-CIT SPECT – image acquisition and pre-processing

^123^I-FP-CIT was intravenously administered in a dose of approximately 185 MBq (specific activity >185 MBq/nmol; radiochemical purity >99%; produced as DaTSCAN™ according to good-manufacturing-practices criteria at GE Healthcare, Eindhoven, The Netherlands). All images were obtained within 3–4 h after injection conforming to Darcourt et al. (2010). ^123^I-FP-CIT has an affinity of Ki = 3.5 nM for DAT and Ki= 10 nM for SERT (Abi-Dargham et al., 1996). We obtained static images for 30 min using a dual-head gamma camera (E.Cam; Siemens, Munich, Germany) with a fan-beam collimator. The images were reconstructed as described earlier (Vriend et al., 2014b), with a Chang's attenuation correction of 0.15 and subsequently reoriented to the anterior-posterior commissure plane in Statistical Parametric Mapping 12 software (SPM 12; Wellcome Trust Center for Neuroimaging, London, UK).

### 2.4 Regions of interest

We used FreeSurfer 6.0 (Athinoula A. Martinos Center for Biomedical Imaging, Boston, MA, USA) with default settings to individually segment Regions of interest (ROIs) from 3D T_1_-weighted MRI scans. These T_1_-weighted scans were acquired at different MRI systems at Amsterdam UMC (location VUmc). See the supplementary material for the scan parameters. We selected the bilateral striatal caudate head and putamen (DAT regions) and the bilateral extrastriatal hippocampus and thalamus (SERT regions) as our ROIs. The putamen was divided into an anterior and posterior part by a line perpendicular to the anterior commissure. Similarly, the head of the caudate nucleus was constrained to voxels anterior to the anterior commissure. All ROIs were visually inspected for segmentation errors (see results).

### 2.5 ^123^I-FP-CIT SPECT and T_1_ co-registration and analysis

^123^I-FP-CIT SPECT scans were co-registered with the T_1_-weighted scans using a previously established method (Joling et al., 2018) in SPM 12. Briefly, hyper-intense FreeSurfer segmentations of the striatal regions were superimposed on the original T_1_-weighted scans to create a common landmark in both the MRI and ^123^I-FP-CIT SPECT scan to allow co-registration based on this mutual spatial information. We calculated binding ratios per subject for each of the striatal and extrastriatal ROIs using the bilateral Crus II of the cerebellum (excluding the vermis) in the automated anatomical labeling (AAL) atlas as a reference (REF). Binding ratios were calculated according to: [(ROI – REF)/REF]. Consistent with previous studies (Joling et al., 2018, 2017; Vriend et al., 2014a, 2014b), we did not perform partial volume correction.

### 2.6 Statistics

Analyses were performed in SPSS 22 (IBM Corp, Armonk, NY, USA). Distributions of the variables were inspected using histograms, Q-Q plots, and Kolmogorov-Smirnov tests. We describe demographics and clinical characteristics using means and standard deviations, unless indicated otherwise. To investigate the association between ^123^I-FP-CIT binding and cognitive performance we performed hierarchical multiple regression. Because of the natural decline in ^123^I-FP-CIT binding with aging, age was entered in the first block of the hierarchical multiple regression analysis. In the second block, age, sex and education adjusted scores on our cognitive tests were *simultaneously* entered in the regression model to investigate the association with ^123^I-FP-CIT binding in the three DAT-rich striatal regions (caudate head and anterior and posterior putamen) and SERT-rich thalamus and hippocampus. We investigated the relation between executive functions, processing speed, attention and working memory and ^123^I-FP-CIT binding in the head of the caudate nucleus, anterior putamen and posterior putamen. Although we did not expect strong associations between cognitive performance and ^123^I-FP-CIT binding in the posterior putamen – which is mainly involved in motor functions – we performed this association as an internal control. All available cognitive test outcomes were fed into the regression model of the thalamus, because the thalamus seems vital for all cognitive processes (Halassa and Kastner, 2017). Memory tests were fed into the regression model with hippocampal ^123^I-FP-CIT binding. This analysis scheme is also outlined in Table 1. To determine the neuropsychological measures that needed to be modeled with ^123^I-FP-CIT binding in the different ROIs and limit the number of tests we applied a backward selection procedure as previously described (Vriend et al., 2016). It is also generally accepted that there is a large overlap between processes underlying executive functions, attention and working memory (McCabe et al., 2010) and the tests designed to measure it, providing additional credence to first perform a backward selection procedure. For the caudate head and anterior and posterior putamen the following cognitive tests were initially entered into the second step of the model (age was entered in the first step): SCWT-I, TMT-A, SCWT interference score, TMT set-shifting score, digit span backward and digit span forward. For the thalamus we additionally entered the delayed recall sore on the RAVLT and RCFT. ^123^I-FP-CIT binding in the hippocampus was modeled with the delayed recall sore on the RAVLT and RCFT only (Table 1). Predictors (i.e. neuropsychological test scores) were removed one-by-one from the model if their regression coefficient (β) fell short of our statistical threshold (i.e. *P*>0.05). Age always remained in the model regardless of significance. The model was re-evaluated after removal of the predictor and repeated until all remaining predictors had a *P*<0.05. This process was performed for each of the five ROIs separately, resulting in a maximum of five final models that needed to be statistically evaluated (see results). The final regression models were bootstrapped using 1000 iterations and we report bias corrected and accelerated (BCa) confidence intervals and *P*-values (*P*_bca_) for a more robust estimate of the association. All assumptions of multiple regression analyses were assessed and met. We corrected the final models for multiple comparisons with Simple Interactive Statistical Analysis (SISA; http://www.quantitativeskills.com/sisa/calculations/bonhlp.htm), an online tool that uses the mutual correlation coefficient between variables (binding ratios in our bilateral ROIs; striatal *r*_mean_ = 0.81) to adjust the statistical threshold and allows a less stringent correction than, e.g. Bonferroni. For the striatal ROIs this resulted in a statistical threshold (*P_corr_*) of *P_corr_*<0.043. As a sensitivity analysis, we additionally added UPDRS-III or dopaminergic medication status to the third step of the models to adjust for these possible confounding variables and performed the regression analyses in the group of medication-naïve PD patients (*N* = 85). To exclude the possible influence of ROI volume we lastly added FreeSurfer-based volume as a nuisance covariate to the third step of the final models.

## 3 Results

### 3.1 Group characteristics

**Fig. 2:**
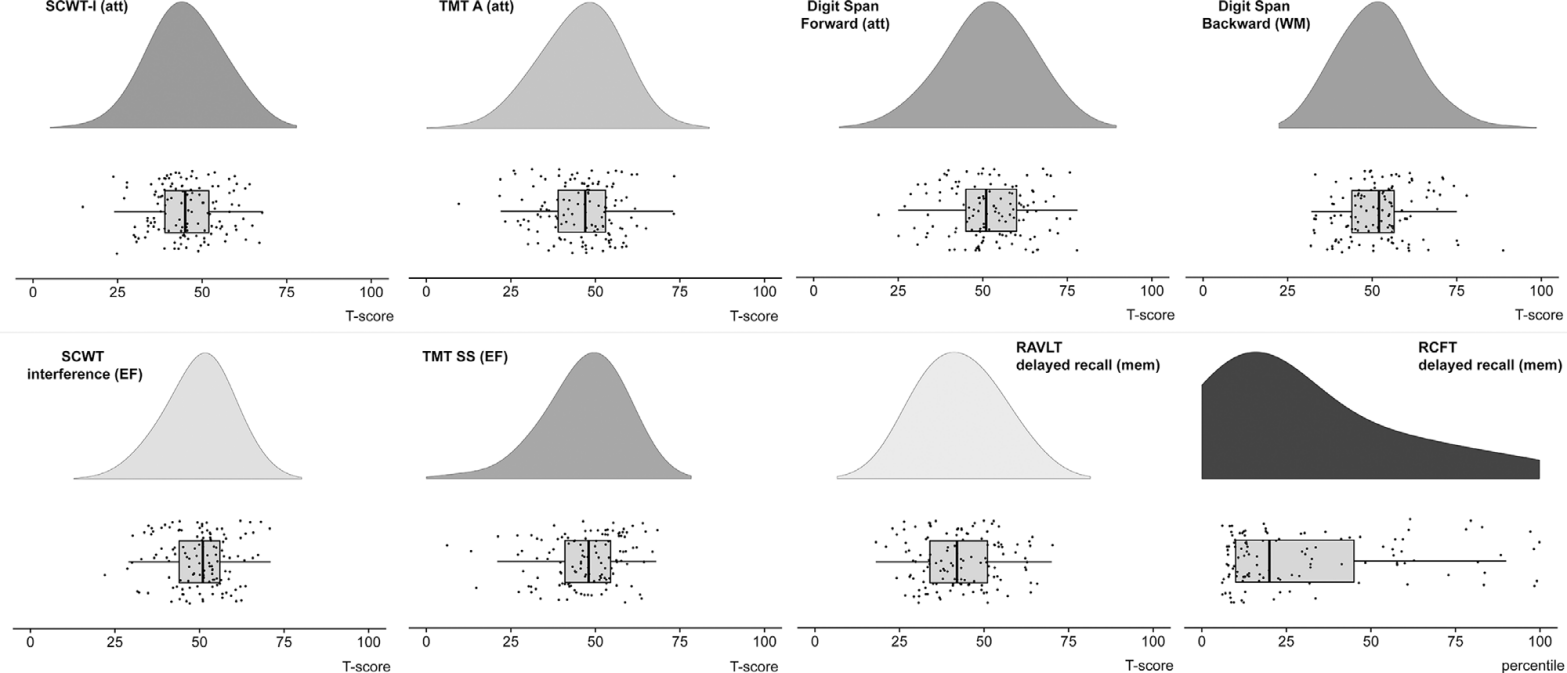
Rainbow plot showing the performance of our sample on the various neuropsychological tests. Scores were converted to standardized *T*-scores (with a mean of 50 and standard deviation of 10) or percentiles to adjust for age, sex and/or educational level, using the appropriate Dutch norm scores (Schmand et al., 2012). Abbreviations: SCWT-I: Stroop Color Word Task card I, TMT A: trail making task part A, TMT SS: trail making task set shifting score, RAVLT: Rey Auditory Verbal Learning Task, RCFT: Rey Complex Figure Task., att = attention, WM = Working memory, EF = Executive functions, mem = memory.

There were 233 PD patients with a ^123^I-FP-CIT SPECT and T1-weighted MRI scan. Of those, a total of 99 were excluded (see flowchart in Fig. 1) leaving 134 PD patients for analysis. Five additional patients had to be excluded due to segmentation failures in FreeSurfer. The final sample thus consisted of 129 PD patients (36.4% female) with a mean age of 65.3(± 10.8). The majority of PD patients (75.2%) had a Hoehn & Yahr stage of ≤2. See Table 2 for further sample characteristics. See Fig. 2 for rainbow plots of the performance on the different neuropsychological tests.

**Table 2:** Sample characteristics.

*N* patients (% female)	129 (36.8)	
Age (years)	65.3 ± 10.8	
UPDRS-III	22.6 ± 9.7	
BDI (*N* = 123)	11.5 ± 7.7	
Education level (in %)		
2	1.6	
3	7.0	
4	16.3	
5	24.0	
6	22.5	
7	28.7	
H&Y stage (in %) *N* = 128		
1	17.8	
1.5	2.3	
2	55.0	
2.5	19.4	
3	4.7	
Subjective disease duration (years)^a^	3.0 ± 3.9	
MMSE	28.3 ± 1.7	
DRT (yes/no)	44/85	
LEDD (mg/day)	266.9 ± 918.0	
**Cognitive tests**	**Mean ± SD [range]**^b^	**Percentage impaired**^c^
SCWT-I	45.2 ± 9.8 [15–68]	4.6%
SCWT interference	49.9 ± 9.2 [22–71]	1.5%
TMT-A	45.6 ± 10.8 [10–73]	8.3%
TMT set-shifting	46.9 ± 11.2 [6–68]	6.9%
Digit span – forwards	51.8 ± 11.6 [19–78]	3.1%
Digit span – backwards	51.7 ± 10.7 [32–89]	0.0%
RAVLT – delayed recall	42.8 ± 11.2 [18–70]	12.9%
Rey figure – delayed recall	30.4 ± 26.9 [10–100]	0.0%

Education level was scored according to the Dutch classification system of Verhage (1964) that ranges from 1 = primary school not finished, to 7 – university or higher.

a Measured from the first sign of motor symptoms.

b All neuropsychological tests were converted to Dutch norm scores to correct for age, sex and/or education level. Scores are presented as *T*-scores, except the Rey figure – delayed recall (percentile).

c Impairment was defined as >2 SD below the mean of the Dutch norm scores. Abbreviations: UPDRS-III: Unified Parkinson's disease Rating Scale part III (motor symptom severity), H&Y = Hoehn & Yahr, MMSE = Mini-Mental State Examination, DRT = dopamine replacement therapy, LEDD = Levodopa Equivalent Daily dose, BDI = Beck Depression Inventory. SCWT-I = Stroop Color Word Task card I, SCWT interference = Stroop Color Word Task interference score, TMT - A = Trail Making Task part A, TMT set-shifting = Trail Making Task set-shifting score, RAVLT = Rey Auditory Verbal Learning Task.

### 3.2 Backward selection procedure

Based on the backward selection procedure the final regression models that needed to be statistically evaluated were the caudate head with SCWT-I and the anterior putamen with SCWT-I and TMT-A. No final models were evaluated for the posterior putamen, and the extrastriatal thalamus and hippocampus (all *β*s with *P*>0.05).

### 3.3 Association between ^123^I-FP-CIT SPECT and cognitive functioning

**Fig. 3:**
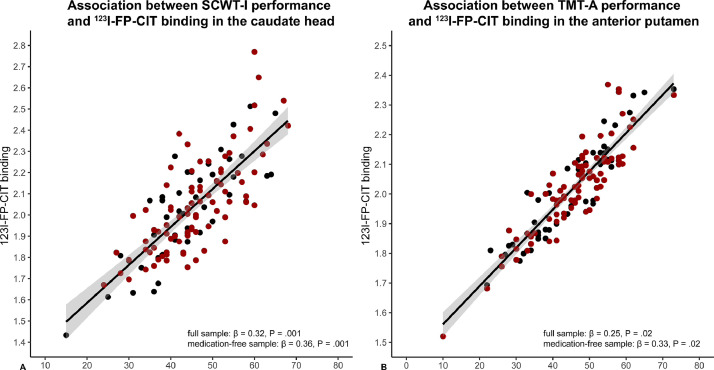
Scatter plots of the associations between cognitive performance and striatal dopamine transporter availability. (A) Positive association between performance on Stroop card I (reading words) and dopamine transporter availability in the caudate head. (B) Positive association between performance on the trail making task part A (connecting consecutively numbered circles) and dopamine transporter availability in the anterior putamen. The black trend line and gray confidence interval are based on the full sample. The red dots in the plot represent the medication-naïve Parkinson patients (*N* = 85). FP-CIT binding is residualized for age. Values on the *x* axis represent standardized *T*-scores (see methods).

Performance on SCWT-I (word reading) showed a significant association with mean ^123^I-FP-CIT binding in the bilateral caudate head (*β* = 0.32, *P*_bca_ = 0.001; Fig. 3(a)) and anterior putamen (*β* = 0.18, *P*_bca_ = 0.033; see Table 3). ^123^I-FP-CIT binding in the anterior putamen additionally showed a positive association with performance on TMT-A (drawing lines between consecutively numbered circles; *β* = 0.25, *P*_bca_ = 0.02; Fig. 3(b)). Adding markers for disease severity (UPDRS-III or Hoehn & Yahr stage) or duration (subjective disease duration), severity of depressive symptoms (BDI) or medication status had no effect on the association between SCWT-I and ^123^I-FP-CIT binding in the bilateral caudate head or between TMT-A and ^123^I-FP-CIT binding in the anterior putamen, although the association between ^123^I-FP-CIT binding in the anterior putamen and SCWT-I performance was no longer significant after adding UPDRS-III (*β* = 0.15, *P*_bca_ = 0.06), Hoehn & Yahr stage (*β* = 0.15, *P*_bca_ = 0.07), BDI (*β* = 0.17, *P*_bca_ = 0.06) or subjective disease duration (*β* = 0.12, *P*_bca_ = 0.17).

**Table 3:** Associations between [123I]FP-CIT binding and cognitive performance.

ROI	MODEL	B (SE)	95% CI (BCa)	BETA	P_BCa_
CAUD	Age	−0.014 (0.004)	−0.022, −0.005	−0.309	0.001
	SCWT-I	0.015 (0.004)	0.009, 0.022	0.312	0**.001**
A-PUT	Age	−0.005 (0.004)	−0.013, 0.003	−0.112	0.237
	SCWT-I	0.009 (0.004)	0.001, 0.017	0.186	0**.038**
	TMT-A	0.010 (0.005)	0.002, 0.020	0.233	0**.032**

For each ROI, age was entered in model 1. Only the results of model 2 are shown here. Abbreviations: CAUD = caudate nucleus, A-PUT = anterior putamen, SE = standard error, CI= confidence interval, BCa = bias corrected and accelerated, SCWT-I = Stroop task card I (reading the words of the colors), TMT-A = trail-making task card A (drawing lines between consecutively numbered circles). *P*-values are bootstrapped.

When performing the analyses in the subgroup of medication-naïve PD patients (*N* = 85) the associations between SCWT-I performance and ^123^I-FP-CIT binding in the anterior putamen was no longer significant (*β* = 0.18, *P*_bca_ = 0.08), while the association between SCWT-I performance and ^123^I-FP-CIT binding in the caudate head (*β* = 0.36, *P*_bca_ =0.001) and TMT-A with ^123^I-FP-CIT binding in the anterior putamen (*β* = 0.33, *P*_bca_ = 0.02) remained and even showed a slight increase in strength. Adding volume of the bilateral ROI as a nuisance covariate had no effect on the significance of these associations.

Finally, because neuropsychological data was missing for *N* = 2 on TMT-B and *N* = 10 on RCFT – which may have impacted the backward selection procedure – we additionally performed the backward selection procedure in the subsample with full neuropsychological data (*N* = 118). This had no effect on the final models that needed to be evaluated, nor their statistical significance.

## 4 Discussion

In this study we investigated the association between striatal and extrastriatal ^123^I-FP-CIT binding and cognitive performance in a large sample of early-stage PD patients. Using bootstrapped multiple regression analysis our main finding was that age-adjusted ^123^I-FP-CIT binding in the head of the caudate nucleus and anterior putamen were positively associated with cognitive performance on the SCWT card I and TMT card A, respectively. These associations remained even after additional correction for markers of disease severity (UPDRS-III, Hoehn & Yahr stage or disease duration) or ROI volume and when the analyses were performed in a subset of medication-naïve PD patients (*N* = 85). Additional post-hoc analysis showed that ^123^I-FP-CIT binding in both the left and right caudate head or anterior putamen contributed approximately equally to the reported associations (data not shown). No associations were found with SERT availability in the thalamus or hippocampus and cognitive performance.

Extensive prior studies have implicated the head of the caudate nucleus and anterior putamen in cognitive processes. Both striatal subregions are involved in the associative CSTC circuit (Groenewegen and Uylings, 2010) that consists of connections between the basal ganglia, thalamus and multiple prefrontal, parietal and temporal brain regions involved in cognitive control (Postuma and Dagher, 2006; Choi et al., 2012). The TMT-A is a measure of visual search and visuomotor speed (Crowe, 1998; Lezak et al., 2012). SCWT-I can be seen a measure of attention and processing speed (Christopher et al., 2014). Because these associations remained significant after adjusting for disease severity and duration, these associations are less likely the result of PD-related decreases in hand speed or speech tempo. Although attention may also be a common denominator of both the TMT-A and SCWT-I, the digit span forward task – a pure attention task – did not explain enough variance to survive the selection procedure. The results are therefore less likely to be related to attention. Instead they may point towards a role of PD-related degeneration of dopamine projections towards the head of the caudate nucleus and anterior putamen in a slowing of processing speed; a fundamental cognitive ability. Of note is that the reported associations were most pronounced in the unmedicated sample, in which the deficiencies of the dopaminergic system leading to slowing of processing speed are not alleviated by dopaminergic medication.

Previous studies have generally shown an association with (*higher-order)* executive functions, using either an aggregated score from a neuropsychological screening battery (Chung et al., 2018) or composite scores of individual tests (Siepel et al., 2014; Nobili et al., 2010). Based in part on these studies and others (Trujillo et al., 2015; Vriend et al., 2015; Bruck et al., 2001; Rinne et al., 2000), we also expected DAT availability in the head of the caudate nucleus and anterior putamen to be related to executive functions. At first glance, our results therefore seem inconsistent with our hypothesis and prior research. Nevertheless, because processing speed is a cognitive process that drives executive functions (Marco et al., 2012; Salthouse, 2005) and both the SCWT and TMT were part of the executive measures of previous studies, it could be that these results are at least partly explained by the shared variance with this fundamental cognitive ability. Indeed, multiple studies have shown an association between dopamine signaling and processing speed in non-PD samples (Beierholm et al., 2013; Eckart and Bunzeck, 2013), although we cannot completely rule out a role for an attentional component. The dissociation between an association of the SCWT-I and TMT-A with the head of the caudate nucleus and anterior putamen, respectively (after correcting for markers of disease severity) may have something to do with the fact that the SCWT-I is a verbal task and the TMT-A requires manual execution. At this point, however, this remains speculative.

In contrast to our hypothesis based on previous research (Chung et al., 2018; Siepel et al., 2014; Nobili et al., 2010; Jokinen et al., 2009), there was no association between executive functions and striatal DAT availability. This might be due to methodological differences. In contrast with the current study, previous studies have not always accounted for age during their analysis of the ^123^I-FP-CIT binding. Because DAT availability declines with natural aging, the guidelines of the European Association of Nuclear Medicine (EANM) recommend adjusting for age to avoid overinterpretation (Darcourt et al., 2010). In fact, when Siepel and colleagues adjusted their results for age, their association between ^123^I-FP-CIT binding and executive functions was no longer significant (Siepel et al., 2014). Furthermore, SSRIs are frequently used in PD (Joling et al., 2018; Vriend et al., 2014c) and significantly increase the quantification of DAT availability by ^123^I-FP-CIT (Booij et al., 2007). In our study we excluded all PD patients on SSRIs for this reason (approximately 10%; see flowchart), while previous studies generally did not report on SSRI use which may have led to differences in findings.

Because of the moderate affinity of ^123^I-FP-CIT binding for SERT, we also investigated the relationship between cognition and ^123^I-FP-CIT binding in the thalamus and hippocampus. Our backward selection procedure, however, did not produce a final model for statistical evaluation. Previous studies using a radiotracer that is selective for SERT, i.e. [^11^C]-DASB, showed lower SERT availability in cortical, limbic and thalamic regions in MCI patients compared with healthy controls and a negative correlation between cortical SERT availability and memory performance (Smith et al., 2017). In PD, MMSE scores were negatively associated with [^11^C]-DASB binding in the caudate, but this was based on a metaregression analysis. (Pagano et al., 2017) Based on these results we hypothesize that ^123^I-FP-CIT may not be sufficiently sensitive to investigate the relationship between the serotonergic system and cognitive functions, even though it was sufficiently sensitive to find a relationship with other PD-related symptoms (Joling et al., 2018, 2017). A study that specifically examines the relation between cognition and SERT using a selective radiotracer in PD is therefore warranted.

Strengths of this study include the large sample size, comprised largely of drug-naïve PD patients, the diverse set of cognitive tests used, and the wide range in cognitive assessments. Furthermore, we performed sensitivity analyses to verify the robustness of the results and excluded patients on drugs that may influence ^123^I-FP-CIT binding. A limitation of the study is that we did not use a radiotracer that is selective for SERT, which may have hampered our ability to detect an association between SERT availability and cognitive function. Furthermore, because not all patients performed two neuropsychological tests per cognitive domain, we were unable to classify their cognitive status according to the relevant diagnostic criteria for PD-MCI level II (Litvan et al., 2012) and no information was available on impairment in daily life to establish PD dementia (Emre et al., 2007).

The absence of a healthy control group also impeded us from determining the specificity of our results for PD. The pathophysiology of PD therefore merely provides a good opportunity to study how (pathological) variation in DAT availability is related to cognitive ability.

In conclusion, using ^123^I-FP-CIT SPECT in a large sample of early-stage PD patients, we observed a positive association between striatal DAT availability and performance on the SCWT-I and TMT-A, likely reflecting processing speed. These results support a role of PD-related dopamine degeneration in this fundamental cognitive ability.

## Funding

None.

## Declaration of Competing Interest

None.

## Appendix Supplementary materials

Image, application 1
