# Supplementary material for: Processing speed is related to striatal dopamine transporter availability in Parkinson's disease
Source: Neuroimage Clin. 2020 Apr 21;26:102257. doi: 10.1016/j.nicl.2020.102257 (PMC7186552; doi:10.1016/j.nicl.2020.102257)
Supplement: Supplementary file 1 [file mmc1.docx]

SUPPLEMENTARY MATERIAL

**MRI Scan parameters:**

- GE Signa HDxT 3.0T (General Electric medical Systems, Milwaukee, WI, USA) with an eight‐ channel head coil (8HRBRAIN). (TR=7.82 ms, TE=3 ms, TI=450 ms; 256x256 mm; voxel size 1.0 mm x 0.977 mm x 0.977 mm; 172 slices)
- GE Discovery* MR750 3.0T (GE medical Systems) with an eight‐channel head coil (8HRBRAIN). (TR=8.21 ms, TE=3.22 ms, TI=450 ms; 265x256 mm; voxel size 1.0 mm x 0.977 mm x 0.977 mm; 176 slices).
- GE Signa HDxt 1.5T (GE medical Systems) with a Head Neck Spine head coil. (TR=12.3 ms, TE=5.2 ms, TI=450 ms; 256x256 mm; voxel size 1.5 mm x 1.0 mm x 1.0 mm; 172 slices)
- Toshiba Vantage Titan 3.0T (Toshiba America Medical Systems Inc., Tustin, CA, USA) with a 32 channel head SPDR coil. (TR=9.5 ms, TE=3.2 ms, TI=800 ms; 256x256 mm; voxel size 1.0 mm x 1.0 mm x 1.0 mm; 176 slices)
- Philips Ingenuity 3.0T (Philips, Eindhoven, the Netherlands) with an eight‐channel SENSE head coil. (TR=7.0 ms, TE=3.0 ms; 288x288 mm; voxel size 1.0 mm x 1.0 mm × 1.0 mm; 180 slices)
